# The effect of acute maximal exercise on postexercise hemodynamics and central arterial stiffness in obese and normal‐weight individuals

**DOI:** 10.14814/phy2.13226

**Published:** 2017-03-31

**Authors:** Kanokwan Bunsawat, Sushant M. Ranadive, Abbi D. Lane‐Cordova, Huimin Yan, Rebecca M. Kappus, Bo Fernhall, Tracy Baynard

**Affiliations:** ^1^ Department of Kinesiology and NutritionUniversity of Illinois at ChicagoChicagoIllinois; ^2^ Department of AnesthesiologyMayo ClinicRochesterMinnesota; ^3^ Department of Preventive MedicineNorthwestern UniversityChicagoIllinois; ^4^ Department of Exercise and Health SciencesUniversity of Massachusetts BostonBostonMassachusetts; ^5^ Department of Health and Exercise ScienceAppalachian State UniversityBooneNorth Carolina

**Keywords:** Arterial stiffness, blood pressure, exercise, obesity

## Abstract

Central arterial stiffness is associated with incident hypertension and negative cardiovascular outcomes. Obese individuals have higher central blood pressure (BP) and central arterial stiffness than their normal‐weight counterparts, but it is unclear whether obesity also affects hemodynamics and central arterial stiffness after maximal exercise. We evaluated central hemodynamics and arterial stiffness during recovery from acute maximal aerobic exercise in obese and normal‐weight individuals. Forty‐six normal‐weight and twenty‐one obese individuals underwent measurements of central BP and central arterial stiffness at rest and 15 and 30 min following acute maximal exercise. Central BP and normalized augmentation index (AIx@75) were derived from radial artery applanation tonometry, and central arterial stiffness was obtained via carotid‐femoral pulse wave velocity (cPWV) and corrected for central mean arterial pressure (cPWV/cMAP). Central arterial stiffness increased in obese individuals but decreased in normal‐weight individuals following acute maximal exercise, after adjusting for fitness. Obese individuals also exhibited an overall higher central BP (*P *<* *0.05), with no exercise effect. The increase in heart rate was greater in obese versus normal‐weight individuals following exercise (*P *<* *0.05), but there was no group differences or exercise effect for AIx@75. In conclusion, obese (but not normal‐weight) individuals increased central arterial stiffness following acute maximal exercise. An assessment of arterial stiffness response to acute exercise may serve a useful detection tool for subclinical vascular dysfunction.

## Introduction

Obese individuals have a higher prevalence of hypertension compared with their normal‐weight counterparts (Fenske et al. 2013; Vaneckova et al. 2014). Central arterial stiffening is associated with incident hypertension and negative cardiovascular outcomes (Cruickshank et al. 2002) and may serve as a potential mechanism underlying the elevated cardiovascular disease risk in obese individuals (DeMarco et al. 2014). Moreover, central arterial stiffness has also been shown to be a key determinant of elevated central blood pressure (BP), another cardiovascular risk factor (Payne et al. 2010). Indeed, obese individuals have higher central BP and stiffer central arteries than normal‐weight individuals (Kolade et al. 2012). This is of clinical importance because increases in central BP, as well as in central arterial stiffness, have shown to be independent predictors of adverse cardiovascular events such as stroke, chronic kidney disease, and hypertension in healthy and clinical populations (Wilson et al. 2004; Chirinos et al. 2005; Mattace‐Raso et al. 2006). Increased central arterial stiffness has also been implicated in the pathology of obesity‐related hypertension (DeMarco et al. 2014).

Acute maximal exercise can induce substantial changes in arterial stiffness and may uncover vascular abnormalities, not apparent at rest (Shim et al. 2011; Yan et al. 2014). Like high‐intensity resistance exercise (Heffernan et al. 2007a), such exercise modality has been shown to transiently increase central arterial stiffness postexercise in young normal‐weight adults (Rakobowchuk et al. 2009; Yan et al. 2014). Such a response is likely driven by the transient increase in sympathetic nerve activation following such high‐intensity exercise (Stuckey et al. 2012), given the independent and positive association between sympathetic nerve activity and central arterial stiffness (Swierblewska et al. 2010). With respect to obesity, only one study reports only higher central pressure wave reflection, measured by augmentation index (AIx) in overweight individuals (~65 years), following maximal exercise despite similar central BP, which may suggest a global increase in central arterial stiffness (Shim et al. 2011). However, central arterial stiffness, measured by central pulse wave velocity (cPWV) did not increase further following exercise in these older overweight adults, likely due to higher baseline cPWV (Shim et al. 2011). Although the modulatory effect of maximal exercise on central arterial stiffness has been demonstrated in young normal‐weight adults (Rakobowchuk et al. 2009; Yan et al. 2014), little information, to date, exists regarding changes in central arterial stiffness and central BP following maximal exercise in obese individuals. Thus, understanding how obesity per se influences central hemodynamics following exercise is of clinical importance, especially because of the increased likelihood for sudden cardiovascular events that may occur following vigorous exertion in susceptible individuals (Albert et al. 2000; Thompson et al. 2007). Specifically, higher central arterial stiffness following maximal exercise in obese individuals may contribute to this risk by increasing left ventricular afterload, depressing coronary perfusion, and potentiating acute cardiovascular events (Hamilton et al. 2007).

The purpose of this study was to evaluate central hemodynamics and arterial stiffness during recovery from acute maximal aerobic exercise in obese and normal‐weight individuals. We hypothesized that obese individuals would exhibit an increased arterial stiffness and central BP following maximal exercise, whereas a decrease in arterial stiffness and central BP would be observed in normal‐weight individuals.

## Methods

### Participants

Sixty‐seven young, healthy volunteers (46 normal‐weight and 21 obese) participated in this study. All participants were free of cardiovascular, metabolic, renal, or respiratory disease and were non‐smokers. None of the participants were taking any medication including over‐the‐counter pain/anti‐inflammatory medication or multi‐vitamin/antioxidant supplement. All participants exhibited normal sinus rhythm and had no history of arrhythmias. Obesity was classified as having a body mass index (BMI) within 30–40 kg/m^2^. All participants were recruited from local community or university population. This study was approved by the Institutional Review Board of the University of Illinois. All participants signed written informed consent prior to participation.

### Study design

We used a cross‐sectional design to test our hypothesis. All participants were at least 3‐h postprandial and did not exercise or consume caffeine or alcohol for 24‐h before reporting to our laboratory. Female participants were tested during days 1–7 of their menstrual cycle or during the placebo phase for those taking oral contraceptives to control for the effects of hormonal fluctuations on arterial stiffness (Robb et al. 2009). Height and weight were obtained using a stadiometer and a balance scale (to the nearest 0.1 cm or kg). Then, participants rested for 10 min in the supine position in a temperature‐controlled room before taking measurements at baseline (before), 15 (Post‐15) and 30 (Post‐30) minutes postmaximal exercise.

### Brachial artery blood pressure assessment

Brachial systolic BP (bSBP) and diastolic BP (bDBP) were obtained using an automated oscillometric cuff (HEM‐907XL, Omron Corporation, Japan). Measurements were made in duplicate, and the average value was used for analysis. If the two values differed by >5 mmHg, a third measure was obtained, and the closest two of the three values were used for analysis. Mean brachial pressure (MAP) was calculated as (SBP–DBP)/3) + DBP.

### Wave reflection and central blood pressure

Radial pressure waveforms were obtained in the supine position using applanation tonometry with a high‐fidelity strain‐gauge transducer (SphygmoCor, AtCor Medical, Sydney, NSW, Australia). Using a generalized validated transfer function (Pauca et al. 2001), central systolic BP (cSBP), diastolic BP (cDBP), central MAP (cMAP) were derived from the central BP waveforms.

Augmentation index normalized to HR of 75 bpm (AIx@75) was derived from contour analysis of the radial pressure waveforms. AIx@75was calculated as the ratio of the amplitude of the pressure wave above its systolic shoulder (i.e., the difference between the early and late systolic peaks of the arterial waveform) to the central pulse pressure, and then normalized to a HR of 75 bpm to minimize an influence from HR (Wilkinson et al. 2000).

### Central pulse wave velocity

cPWV was measured based on established guidelines (Van Bortel et al. 2002). A high‐fidelity strain‐gauge transducer (Millar Instruments, Houston, TX) was used to obtain pressure waveforms for a 10‐sec epoch from the right common carotid artery and the right femoral artery. The peak of an in‐phase R wave, as obtained from sequential electrocardiogram (ECG) monitoring (CM_5_ configuration) was used as a timing marker. The ECG recordings were also used to obtain baseline and recovery HR. The foot of the pressure wave was identified automatically, removing potential observer bias, using an algorithm that detects the initial upstroke via a line tangent to the initial systolic upstroke point of the pressure tracing and an intersecting horizontal line through the minimum point. This algorithm has been shown to be highly reproducible (Chiu et al. 1991). The distances from the carotid artery to the suprasternal notch and from the suprasternal notch to the femoral artery were measured as straight lines using a tape measure. The distance from the carotid artery to the suprasternal notch was then subtracted from the suprasternal notch‐femoral artery segment to correct for differences in propagation direction along the arterial path length and taken as a measure of central arterial stiffness. The tape measure was held in a straight line above the stomach to avoid wrapping or touching the measurement tape on the subject's stomach before taking the distance. Integral software assessed the pulse wave quality (strength of the pulse wave signal, pulse height variation, pulse length variation, and baseline variation) and SD of mean time differences (SphygmoCor, AtCor Medical, Sydney, Australia). This technique has been shown to be highly reproducible (Wilkinson et al. 2002).

### Peak oxygen consumption

Peak oxygen consumption (*V*O_2peak_) was measured using a breath‐by‐breath metabolic system (Quark b^2^, Cosmed, Rome, Italy) during an incremental graded cycling exercise test until exhaustion (Excaliber Sport, Lode, the Netherlands). The participants began with a 1‐min of warm‐up with no resistance. The first workload was set at 50 W and gradually increased by 30 W every 2 min until test termination. The participants pedaled at a cadence ≥60 rpm. Ratings of perceived exertion (RPE) were assessed once per stage. The test was terminated when participants met three of the following five criteria: (1) RPE score of 17 or greater on the on the Borg scale (scale 6–20), (2) respiratory exchange ratio of at least 1.1, (3) no change in HR within a change in workload, (4) a “plateau” (increase of ≤150 mL) in *V*O_2_ with an increase in workload, and/or (5) volitional fatigue. Following test termination, the recovery protocol began with a 2 min of light cycling (0W, 50 rpm), followed by a minute of quiet sitting on the cycle ergometer. Participants were then immediately transferred to a table and rested in the supine position for the measurements at 15 and 30 min postexercise (Post‐15 and Post‐30, respectively).

### Statistics

Descriptive characteristics and baseline differences were compared between groups using an independent *t* test. Data were checked for normality of distribution using the Shapiro–Wilk tests. Natural logarithmic transformation was performed on non‐normally distributed variables before statistical analysis. A 2 × 3 ANOVA with repeated measures [group (normal‐weight vs. obese) by time (baseline, post‐15, post‐30)] was conducted on all dependent variables to compare group differences in response to exercise. When a significant group‐by‐task interaction was detected, between‐group differences at each level were examined using appropriate post hoc analyses (Bonferroni correction and independent *t* tests). We also accounted for a group difference in fitness (HR_peak_, cPWV, and cPWV/cMAP variables) and or central BP (cPWV variable only) in the statistical analysis using ANCOVA controlling for *V*O_2peak_ and cMAP. Values are mean ± SE. Alpha was set at *P *<* *0.05. All data were analyzed using SPSS (v 21.0, IBM SPSS, Inc., Armonk, NY).

## Results

Descriptive characteristics are shown in Table 1. Compared with the normal‐weight individuals, obese individuals had higher weight and BMI and a lower *V*O_2peak_ and RER_peak_ (*P *<* *0.05).

**Table 1:** Descriptive characteristics for normal‐weight and obese individuals

	Normal‐Weight (*n* = 46)	Obese (*n* = 21)
Sex (male/female)	21/25	12/9
Race (AA/CA)	9/12	7/5
Age (years)	24 ± 1	24 ± 1
Height (cm)	170.0 ± 1.5	169.6 ± 2.2
Weight (kg)	65.4 ± 1.5	94.0 ± 3.7^a^
BMI (kg/m^2^)	22.5 ± 0.3	32.6 ± 0.4^a^
*V*O_2peak_ (mL/kg/min)	39.3 ± 1.6	30.0 ± 1.4^a^
Test duration (sec)	607 ± 36	657 ± 67
HR_peak_ (bpm)	184 ± 2	190 ± 4
RER_peak_	1.16 ± 0.01	1.12 ± 0.01^a^
Peak wattage (W)	178 ± 8	187 ± 14

Values are mean ± SE. AA, African American; CA, Caucasian; BMI, body mass index; VO_2peak_, peak aerobic capacity; HR_peak_, peak heart rate; RER_peak_, peak respiratory exchange ratio.

a Group difference (*P *<* *0.05).

**Figure 1:**
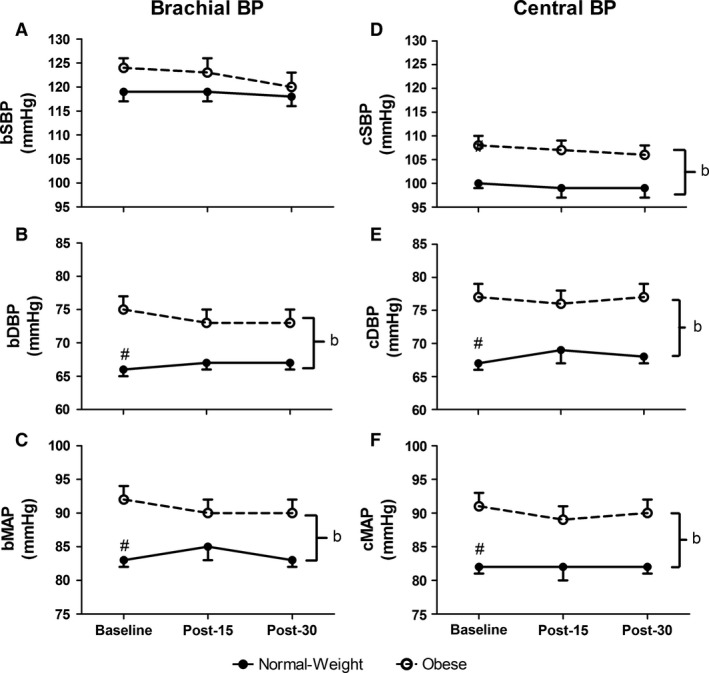
Brachial (A–C) and central (D–F) blood pressure (BP) variables at baseline and at 15‐ and 30‐min postmaximal exercise. bSBP, brachial systolic blood pressure (Panel A); bDBP, brachial diastolic blood pressure (Panel B); bMAP, brachial mean arterial pressure (Panel C); cSBP, central systolic blood pressure (Panel D); cDBP, central diastolic blood pressure (Panel E); cMAP, central mean arterial pressure (Panel F). ^#^group difference at baseline (*P *<* *0.05); ^b^main effect of group (*P *<* *0.05). Values are mean ± SE.

**Figure 2:**
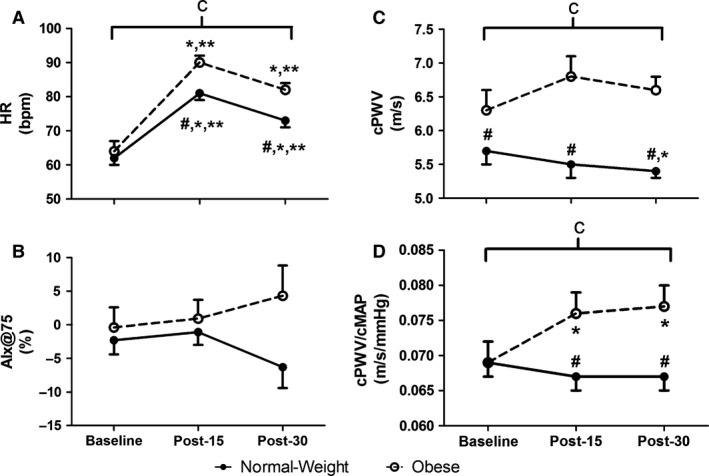
Heart rate (HR, Panel A), augmentation index normalized to a HR of 75 bpm (AIx@75, Panel B), central pulse wave velocity (cPWV, Panel C), and central pulse wave velocity normalized to central mean arterial pressure (cPWV/cMAP) at 15‐ and 30‐min postmaximal exercise. ^#^group difference at baseline (*P *<* *0.05); *different from baseline (*P *<* *0.05); **different from 15‐min postmaximal exercise (*P *<* *0.05); ^a^main effect of time (*P *<* *0.05); ^b^main effect of group (*P *<* *0.05); ^c^interaction effect (*P *<* *0.05). Values are mean ± SE.

Hemodynamic variables are shown in Figures 1A–F and 2A. At baseline, obese individuals exhibited higher baseline bDBP, bMAP, cSBP, cDBP, and cMAP than normal‐weight individuals (*P *<* *0.05, Fig. 1B–F). Following exercise, HR increased from baseline similarly in both groups, but was overall higher in the obese individuals, even after controlling for the effects of fitness and central BP (*V*O_2peak_ and cMAP) (*P *<* *0.05, Fig. 2A). No exercise responses were observed bDBP, bMAP, cSBP, cDBP, and cMAP, but these variables were overall higher in obese individuals versus normal‐weight individuals (*P *<* *0.05 for group, Fig. 1B–F). No main effects of group or time were found for bSBP.

Central arterial stiffness and wave reflection variables are shown in Figure 2C–D. At baseline, obese individuals exhibited higher baseline cPWV than normal‐weight individuals (*P *<* *0.05, Fig. 2C). No main effects of group or time were found for AIx@75. However, there was an interaction for cPWV (*P *<* *0.05, Fig. 2C), which remained significant even after statistically covarying the effects of fitness and central BP (*V*O_2peak_ and cMAP). Following exercise, cPWV decreased at Post‐30 only in the normal‐weight group (*P *<* *0.05, Fig. 2C), but was lower than that in the obese individuals at all time points (*P *<* *0.05, Fig. 2C). Additionally, there was an interaction for cPWV/cMAP (*P *<* *0.05, Fig. 2C, D), which remained significant even after statistically covarying the effects of fitness (*V*O_2peak_). Following exercise, cPWV/cMAP increased at Post‐15 and Post‐30 only in the obese individuals (*P *<* *0.05), and was higher than that in the normal‐weight individuals at Post‐15 and Post‐30 (*P *<* *0.05).

## Discussion

The main findings of this study were that obese individuals exhibited increased central arterial stiffness (cPWV and cPWV/cMAP) compared with normal‐weight individuals following exercise. To the best of our knowledge, this study is the first to evaluate hemodynamics and central arterial stiffness following maximal exercise in young, otherwise healthy obese individuals.

### Obesity, hemodynamics, and arterial stiffness at rest

Obese individuals have elevated cPWV, brachial and central BP under resting conditions as compared with normal‐weight counterparts (Kolade et al. 2012; Kappus et al. 2014). We also observed higher resting brachial (DBP and MAP) and central BP (SBP, DBP, and MAP) and cPWV in obese individuals compared with normal‐weight individuals. Our findings suggest alterations in arterial properties with increased body mass. We also reported no group differences in resting HR or AIx@75, which is consistent with previous studies (Tabara et al. 2008; Shim et al. 2011). In other studies, peripheral wave reflections have been reported to be either lower (Maple‐Brown et al. 2005; Otsuka et al. 2009) or higher (Wykretowicz et al. 2007) with obesity; however, these participants were older than ours, which might account for our different findings.

### Obesity, hemodynamics, and arterial stiffness following exercise

Acute maximal exercise has been shown to induce substantial changes in arterial stiffness and hemodynamics and may unmask vascular abnormalities not seen at rest (Shim et al. 2011; Yan et al. 2014). Little information exists regarding changes in arterial stiffness and hemodynamics following maximal exercise in obese individuals. In this study, we observed no acute maximal exercise effect on BP in either group; however, brachial and central BP (DBP and MAP) were overall higher in obese individuals than in normal‐weight individuals, consistent with the notion of obesity‐related BP elevations (Kappus et al. 2014). HR increased following exercise as expected, but was overall higher in the obese individuals than in the normal‐weight individuals throughout the recovery period (main effect of group). The overall higher HR may suggest augmented sympathetic neural outflow to the heart (Coote 2010).

Acute high‐intensity exercise has been shown to reduce the magnitude of wave reflections in healthy normal‐weight individuals (Casey et al. 2008), suggesting exercise‐induced reduction in arterial stiffness, ventricular dynamics and/or peripheral vasodilation. However, we did not observe any change in AIx@75 following exercise for either group with no group differences in contrast to one study that reported higher global wave reflections immediately following peak bicycling exercise in overweight individuals (Shim et al. 2011). The difference in findings is likely due to age and obesity status differences, such that our participants were young (~24–25 years) and obese, whereas their participants were older (~65 years) and overweight. A previous study suggests that an increase in AIx may not be attributable to increased central arterial stiffness, but rather were principally related to an increase in peripheral resistance, which ultimately affects temporal overlap between forward and reflected wave (Mitchell et al. 2005). Thus, the higher global wave reflections observed in the previous study (Shim et al. 2011) may merely suggest an increase in peripheral resistance and not central arterial stiffness in older overweight adults.

cPWV is regarded as a gold standard for central arterial stiffness assessment (Van Bortel et al. 2012), and cPWV has been shown to transiently increase at 10‐min following exercise (Melo et al. 2016) and then decrease below baseline at 30‐min following exercise (Yan et al. 2014). In the present study, central arterial stiffness increased in the obese participants, but decreased in the normal‐weight individuals following exercise. Our findings may suggest a delay in recovery of maximal exercise‐induced arterial stiffening and/or the lack of exercise‐induced improvement in arterial distensibility in obese individuals. Transient exertion‐induced increases in central arterial stiffness have also been documented following acute firefighting (Fahs et al. 2011), heavy resistance exercise (Heffernan et al. 2007a), and acute sprint interval exercise (Rakobowchuk et al. 2009).

Mechanisms underlying acute maximal exercise‐induced central arterial stiffening in obese individuals are unclear. Increases in sympathetic nerve activity have been associated with central arterial stiffening (Hart et al. 2013; Smith et al. 2015), and obese individuals have demonstrated increases in sympathetic nerve activity at rest and exaggerated sympathetic reactivity to stress (Kuniyoshi et al. 2003; Park et al. 2012; Huber and Schreihofer 2016). Obesity‐related sympathetic overactivity has also been attributed to oxidative stress (Smith and Minson 2012), which increases postexercise in obese individuals (Vincent et al. 2004), thus possibly contributing to central arterial stiffening (Chen et al. 2012). Additionally, oxidative stress may induce central arterial stiffening in obesity by decreasing nitric oxide bioavailability and endothelial dysfunction (Perticone et al. 2001; Anderson 2006). Thus, while we did not obtain markers of sympathetic nerve activity, endothelial function, and oxidative stress for this study, it may be reasonable to speculate that sympathetic overactivity, coupled with reduced vasodilatory capacity may explain the increased central arterial stiffening following maximal exercise in this study. The transient increase in central arterial stiffness may also result from excessive vascular wall stress during high‐intensity exercise due to the higher intramuscular pressure during forceful muscular contractions in obesity (Montero et al. 2013, 2014). Since we did not observe any significant change in blood pressure following exercise, and we corrected cPWV for blood pressure, blood pressure per se did not account for the different cPWV responses in the obese individuals.

### Clinical perspectives

Our study is of clinical importance, because we demonstrated exercise‐induced central arterial stiffening even in young, otherwise healthy obese adults. Obese individuals who exhibited central arterial stiffening, especially following strenuous exercise, may be at an increased risk for sudden cardiovascular events as a result of increased left ventricular afterload and depressed coronary perfusion induced by central arterial stiffening (Hamilton et al. 2007). Most importantly, increased arterial stiffness may chronically expose organs, such as the brain and kidneys, to high pulsatile pressure and subsequently lead to pathogenic arterial remodeling and microcirculatory damage, which may ultimately increase cardiovascular disease risk such as stroke and hypertension (Lee and Oh 2010).

### Limitations

There are several limitations to the study: (1) measurements of central arterial stiffness and arterial waveforms were made using non‐invasive techniques, although cPWV is widely accepted as a gold standard measure of central arterial stiffness (Townsend et al. 2015), and central arterial waveforms derived via transfer function has also been validated with invasive techniques (Sharman et al. 2006); (2) we did not obtain measurements immediately following peak exercise and therefore could not detect the magnitude of increase in cPWV from baseline; (3) we did not obtain measures of central obesity and could not conclude if our results were based on overall obesity or central obesity; moreover, obesity was based on BMI classification alone, which is not reflective of actual body composition; (4) there could be a potential racial difference between groups, which could drive the divergent responses in the recovery (Heffernan et al. 2007b; Yan et al. 2014), but this was not the main focus of the current study; (5) we only recorded recovery data up until 30 min postexercise and therefore could not detect how long it would take for central arterial stiffness to return to baseline values for both groups; (6) we did not have information on *V*O_2_ expressed relative to kg of fat‐free mass (No body composition analysis); (7) we did not obtain measures of sympathetic nerve activity, oxidative, or endothelial function for this study, which would have helped better interpret our findings. However, we speculate these factors may be implicated in the stiffening of central arteries following maximal exercise (Park et al. 2012; Smith and Minson 2012; Smith et al. 2015).

## Conclusions

Obese individuals exhibited an increase in central arterial stiffness following acute maximal exercise, a response that remained after expressing stiffness relative to BP and after statistically covarying for fitness. The increase in central arterial stiffness postexercise in obese individuals may precipitate detrimental changes in coronary blood flow and acutely increase risk for sudden cardiovascular events. Therefore, assessing the vascular responses to vigorous exercise may serve as a means to detect tool for subclinical vascular dysfunction and identify those at risk of postexercise sudden cardiovascular events.

## Conflict of Interest

There are no conflicts of interest.
