# Erratum

**DOI:** 10.14814/phy2.13268

**Published:** 2017-04-18

In Bunsawat et al. (2017), the following errors were published in the article.

The last line of the Abstract, “An assessment of arterial stiffness response to acute exercise may serve a useful detection tool for subclinical vascular dysfunction” should instead read “An assessment of arterial stiffness response to acute exercise may serve as a useful detection tool for subclinical vascular dysfunction.”

In the Limitations section, the line “and central arterial waveforms derived via transfer function has also been validated” should instead read “and central arterial waveforms derived via transfer function have also been validated.”

In the last sentence of the Conclusion, the line “the vascular responses to vigorous exercise may serve as a means to detect tool for subclinical vascular dysfunction” should instead read “the vascular responses to vigorous exercise may serve as a detection tool for subclinical vascular dysfunction.”

We apologize for the errors.
